# Young People’s Barriers and Facilitators of Engagement with Web-Based Mental Health Interventions for Anxiety and Depression: A Qualitative Study

**DOI:** 10.1007/s40271-024-00707-5

**Published:** 2024-07-13

**Authors:** Thi Quynh Anh Ho, Lidia Engel, Glenn Melvin, Long Khanh-Dao Le, Ha N. D. Le, Cathrine Mihalopoulos

**Affiliations:** 1 Deakin University, Institute for Health Transformation, Deakin Health Economics, School of Health and Social Development, 221 Burwood Highway, Burwood, VIC 3125 Australia; 2 Monash University Health Economics Group, School of Public Health and Preventive Medicine, Monash University, Melbourne, VIC Australia; 3 School of Psychology, Deakin University, Melbourne, VIC Australia

## Abstract

**Background:**

The prevalence of anxiety and depressive symptoms in young people have increased in many countries around the world. Web-based mental health interventions (or W-MHIs) have the potential to reduce anxiety and depression symptoms for young people. Although W-MHIs have become more widely used by young people since the coronavirus disease 2019 (COVID-19) pandemic, real-world engagement in these W-MHIs has remained low compared with engagement reported in research studies. Moreover, there are limited studies examining factors influencing engagement with W-MHIs in the post-COVID-19 pandemic years.

**Objective:**

This study aims to explore barriers and facilitators of engagement with W-MHIs for anxiety and depression among young people.

**Method:**

Seventeen semi-structured interviews and one focus group with three participants were conducted online via Zoom between February and March 2023. Participants were young people aged 18–25 years who had self-reported experience of anxiety and/or depression in the past 6 months, lived in Australia, and considered using W-MHIs to manage their anxiety and/or depression symptoms. Inductive thematic analysis was performed to understand the key barriers and facilitators of young people’s engagement with W-MHIs.

**Results:**

Both individual- and intervention-related factors influenced young people’s engagement with W-MHIs. Facilitators of engagement included personal trust and beliefs in W-MHIs, ability to contact a health professional, programme suitability (e.g., affordability, content aligning with user needs), programme usability (e.g., user interface), and accessibility of the online platform. Barriers included concerns about online security, lack of human interaction and immediate responses from health professionals (if any), and negative experience with mental health programmes. Participants expressed greater willingness to pay if they could contact health professionals during the programme.

**Conclusion:**

Better promotion strategies for mental health and W-MHI awareness are needed to increase the perceived importance and priority of mental health interventions among young people. Young people should be involved in the W-MHI co-design to enhance the programme suitability and usability for young people, fostering their engagement with W-MHIs.

**Supplementary Information:**

The online version contains supplementary material available at 10.1007/s40271-024-00707-5.

## Key Points for Decision Makers

**Table:** 

Barriers and facilitators of engagement with web-based mental health interventions (W-MHIs) exist at both individual and intervention levels.
Stigma surrounding mental health can influence how young people engage with W-MHIs.
Health professional contact is important and can influence young people’s willingness to pay for W-MHIs.
Increased mental health promotion strategies and de-stigmatising campaigns are needed to enhance young people’s perceptions of mental health and treatments.
Young people should be involved in the co-design process for future W-MHI development.

## Introduction

Nearly one in every eight (13%) young people aged 10–24 years in the world live with a mental disorder [1]. Young people aged 18–24 years are nearly twice as likely as teenagers to experience anxiety and depression [2]. In Australia, the anxiety and depression prevalence rates in young people have grown compared with all other ages [3, 4]. Furthermore, the onset of anxiety and depression usually occurs in early adulthood and individuals with untreated anxiety or depression are at risk of having any mental disorder later in their lifetime [5]. Facilitating access to mental health support is crucial for the early prevention or treatment of underlying conditions in young people.

Digital psychological treatments, such as web-based mental health interventions (or W-MHIs), have become an important alternative to face-to-face mental health services [6]. In this study, W-MHIs refer to online interventions or programmes that are delivered via websites and can be accessed via a computer, laptop, tablet, or smartphone. W-MHIs may be appropriate for those at risk for a mental health disorder or having mild-to-moderate symptoms [7]. W-MHIs have been increasingly utilised among young people due to their feasibility, acceptability, and potential effectiveness in reducing anxiety and depression symptoms [8].

Treatment engagement consists of behavioural (e.g., attendance at treatment sessions) and attitudinal elements (e.g., expectation about treatment helpfulness) [9]. Greater engagement can be associated with post-intervention improvement in mental health [10]. Despite the potential of W-MHIs in improving mental wellbeing, suboptimal engagement levels may limit intervention benefits [11]. Engagement with W-MHIs has been shown to be typically lower than that in research studies [12]. Nearly 40% of participants withdrew before completing at least 25% of all treatment modules in self-guided W-MHIs and more than 83% of participants withdrew before completing the entire programmes [13]. Web-based cognitive behavioural therapy for anxiety and depression reported an average completion rate of 57% for all treatment modules [14]. Low engagement can be a barrier to adopting W-MHIs in healthcare settings and translating intervention benefits into real-world outcomes.

Barriers/facilitators of accessing professional mental health support and user engagement with digital mental health interventions (including W-MHIs) have been investigated among young people with anxiety and depression. Barriers/facilitators can be related to individuals (e.g., perceptions of mental health help-seeking) and interventions (e.g., non-appealing interfaces) [15–18]. Previous research has largely focused on user experience with specific interventions or broader perceptions of digital mental health care, capturing factors influencing initial uptake but potentially overlooking those influencing continued use of digital mental health interventions, particularly for W-MHIs [15–18]. In order to improve engagement (including initial uptake and continued use), it is crucial to explore the experiences of past and current W-MHI users, fostering understanding of barriers/facilitators of engagement with these interventions beyond research context. Notably, the majority of studies on factors influencing user engagement have been conducted prior to the COVID-19 pandemic. Research has identified changes in user engagement behaviour with digital mental health services among young people during the pandemic, such as decreased engagement in ad hoc chats with practitioners [19]. Thus, barriers/facilitators of young people’s engagement with W-MHIs may change over time with W-MHIs becoming better known and utilised by young people. To date, limited research has specifically focused on the engagement with W-MHIs for anxiety and depression among young people after the COVID-19 pandemic. Such knowledge could inform effective strategies to improve user engagement with W-MHIs for better outcomes.

This qualitative study aims to identify factors that influence user engagement with W-MHIs for anxiety and depression in Australia. Young people with previous experience with W-MHIs or an intention to use them were chosen to explore relevant barriers and facilitators that influence their continued engagement with or intention to start using W-MHIs through ethnographic research [20]. Findings from this study can inform the development of programmes or policies to improve engagement with W-MHIs in young people.

## Methods

### Participants

We adopted a purposive sampling strategy, which is considered typical best practice in sampling design for qualitative research [21]. With this strategy, we selected participants who had previously used or known about W-MHIs so that participants could reflect on their experience with W-MHIs. Participants were recruited via social media platforms, including Facebook, X (formerly known as Twitter), LinkedIn, Reddit, and TikTok. Online advertisements contained an expression of interest form (created by Qualtrics [Qualtrics, Provo, UT, USA]). Potential participants indicated interests by providing their email addresses in the form. [First author] emailed interested participants to arrange an individual Zoom call (less than 5 min) to check eligibility. During the call, participants were asked five screening questions. Participants were eligible if they were (1) aged between 18 and 25 years; (2) lived in Australia; (3) were able to speak English; (4) self-reported to experiencing depression and/or anxiety in the past 6 months; and (5) had previously used (or considered using) a W-MHI for depression and/or anxiety. Given that our study targeted young people with mild-to-moderate symptoms, those having been admitted to hospital for mental health treatment in the past month were excluded.

Of 76 participants who expressed interest, 48 were lost to follow-up due to not answering the Zoom calls, resulting in 28 being screened for eligibility, with 20 ultimately participating in the online interviews/focus groups. Four were eligible but did not participate in the interview/focus groups for unknown reasons, and the remaining four were excluded due to being over 25 years of age (*n* = 1), not having used a W-MHI (*n* = 1) and having been admitted to hospital for mental health treatment in the past month (*n* = 2). All participants provided online informed consent via the Qualtrics platform. Following this, participants completed an online questionnaire about demographics and level of anxiety and depression using the Generalised Anxiety Disorder 7-item (GAD-7) scale [22] and Patient Health Questionnaire-8 (PHQ-8) [23]. Participants could choose to participate in a one-on-one interview or focus group depending on what was most convenient for them. Participants who scored ≥ 10 in either the GAD-7 or PHQ-8 were deemed at risk of generalised anxiety disorder or depressive disorder [22, 23] and received information about mental health support services via email after the interview/focus group concluded. Once the consent and demographic information was collected, [first author] sent email invitations to eligible participants for the online interview/focus group. The study received ethics approval (2022-119) from Deakin University Human Research Ethics Committee.

### Data Collection

Online semi-structured interviews and focus groups were conducted to enable an in-depth discussion of contemporary barriers/facilitators, allowing participants to fully express themselves [24]. Given the nature of focus groups, we did not return the interview transcript to participants for review after the interview/focus group concluded. Interview guides were developed based on the extension of unified theory of acceptance and use of technology (UTAUT 2) [25]. The interview schedule is outlined in Online Resource 1.

Interviews were piloted with one young person, one mental health clinician, and one mental health researcher in Melbourne, VIC, Australia. Interviews/focus groups were conducted from February to March 2023 via Zoom by the first author (a 27-year-old female PhD candidate). Before this study, [first author] received interview skills training from a qualified mental health clinician [third author]. [First author] had no prior experience with W-MHIs and was unfamiliar with the participants before the study commenced. Thus, [first author] had few biases and assumptions related to the research topic.

During the interview/focus group, participants could turn on (off) the webcam if they wished to. [First author] took notes during the interview/focus group. All interviews/focus groups were video-recorded using Zoom. The interviews/focus groups consisted of two parts. The first part primarily focused on factors influencing participants’ engagement with W-MHIs. To identify important factors (or attributes) influencing user engagement with W-MHIs, in the second part, we described 12 intervention attributes (e.g., means of therapist contact) and corresponding levels (e.g., contact via chat/email, audio, and video call) using Miro App (www.miro.com) (Online Resource 1). The intervention attributes and levels were developed based on the current literature [15, 17] and data iteration during the interviews/focus groups. Participants shared insights on how these attributes influenced their uptake or continued use of W-MHIs. These attributes could be considered as barriers (or facilitators) if they impeded (or encouraged) the engagement with intervention. Upon the completion of the interview/focus group, each participant received an AU$20 gift card in acknowledgement of their time.

### Sample Size Justification

Preliminary data analysis was performed concurrently with data collection, allowing us to determine when saturation, the point at which ‘additional data do not lead to any new emergent themes’ [26], was reached and recruitment was ceased. More than 85% of the codes emerged after the first eight interviews. Consistent with previous literature [27], nearly all codes (or subthemes) were developed after 13 interviews. No new codes (or subthemes) were found after 17 interviews. Three additional interviews were conducted, resulting in no new code (or subtheme). This suggests that data saturation was reached after 20 interviews (Online Resource 2), enhancing the rigour of our qualitative study [28].

### Data Analysis

Interviews/focus groups were auto-transcribed by Zoom. To increase the study credibility, all the transcripts were checked for accuracy [28] and de-identified by [first author] prior to importing into NVivo version 12 [29]. Inductive thematic analysis was performed by [first author] classifying the themes and subthemes, following six steps as described by Braun and Clarke [30]. An inductive approach was used to derive findings from the dominant themes inherent in raw data [31]. Data analysed by [first author] were then reviewed and discussed with all authors to reach consensus for each identified theme.

First, [first author] read through each of the interview/focus group transcripts to familiarise themselves with the dataset and highlighted all quotes relevant to the study objective. Next, [first author] assigned initial notes to each relevant content. Following this, all initial notes with similar meanings were categorised into specific codes (i.e., semantic level). Labels of codes were named based on barrier/facilitator subthemes identified in a previous systematic review [15, 17] and the interview guide. The semantic codes with similar concepts were compiled and collated to develop initial subthemes. With 12 subthemes developed from the dataset, common meanings among the initial subthemes were identified and categorised into four initial themes. To enhance the rigour and trustworthiness of the research, [second author] reread all codes, subthemes, and themes, and made modifications where relevant. All authors discussed the initial themes and corresponding subthemes and reached an initial consensus. Following this discussion, [first author] reread all the data and checked the consistency of initial themes and subthemes across the dataset. The four initial themes were then refined and renamed upon this checking. The qualitative research was reported in accordance with the COnsolidated criteria for REporting Qualitative research (COREQ) checklist [32].

## Results

There was a total of 17 individual interviews and one focus group with three participants. The interviews/focus groups lasted between 60 and 110 min (mean 78.5 min). Participant characteristics are summarised in Table 1. Participants’ age ranged from 18 to 25 years (mean 22.75 years, standard deviation [SD] 1.68). The majority of participants were males (75%), living in eastern coastal mainland states of Australia (90%), and had a tertiary education (55%). All participants had used at least one W-MHI, with 15% using such interventions for improving general mental wellbeing and 85% using them for managing anxiety or depression. More than half of participants (55%) used W-MHIs developed based on cognitive behavioural therapy or acceptance and commitment therapy.

**Table 1:** Participant characteristics (*N* = 20)

	*n*	%
Mean age, years	22.75	SD 1.68
Male	15	75
Location		
New South Wales	8	40
Queensland	6	30
Victoria	4	20
Western Australia	2	10
Education attainment		
Year 12	4	20
Vocational education and training (e.g., diploma, certificate)	5	25
Higher education (e.g., bachelor’s degree)	11	55
Employment status		
Employed	16	80
Unemployed	4	20
GAD-7 score		
Minimal	3	15
Mild or moderate	16	80
Severe anxiety	1	5
PHQ-8 score		
Minimal	2	10
Mild or moderate	15	75
Moderately severe	3	15
Previous experience with mental health professionals		
Yes	9	45
No	11	55
Previous experience with online mental health programmes		
Web-based programmes (e.g., This Way Up, Mindspot)	16	57
App-based programmes (e.g., Happify)	5	18
Chat-based therapies (e.g., headspace)	5	18
Information websites (e.g., beyondblue)	2	7

*SD* standard deviation, *GAD-7* Generalised Anxiety Disorder 7-item, *PHQ-8* Patient Health Questionnaire-8

**Fig. 1:**
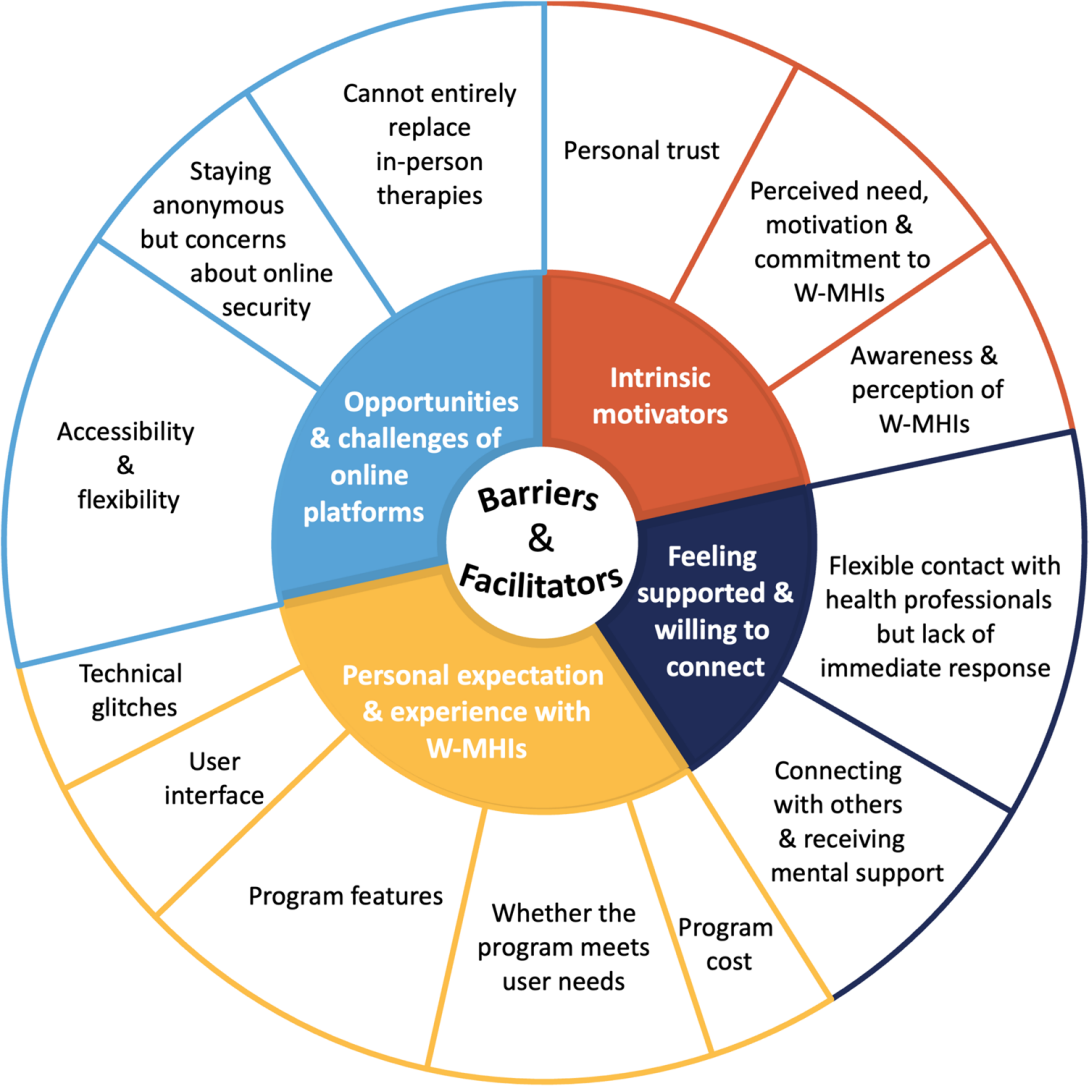
Themes and subthemes of barriers/facilitators of engagement with web-based mental health interventions. *W-MHIs* web-based mental health interventions

Barriers/facilitators included factors related to both individuals and interventions. Four main themes were identified and are described in Fig. 1.

### Theme 1: Intrinsic Motivators

Intrinsic motivators refer to internal factors that can drive individuals’ behaviours to engage with W-MHIs (or discontinue using W-MHIs). These motivators can be personal trust, perceived need and commitment to W-MHIs, personal awareness and perception of W-MHIs.

#### Personal Trust About Web-Based Mental Health Interventions (W-MHIs)

Participants discussed their trust in W-MHI reliability and effectiveness as a potential facilitator of initial uptake. Young people tended to use a W-MHI if it was funded by a reputable organisation (e.g., headspace), advertised on a verified page, and appeared to be popular (e.g., interventions with a great number of users, or those listed in top search results). Some would be more likely to use a W-MHI if referred by trusted people (e.g., family members and friends), especially those having previous experience with the intervention. Other facilitators included user reviews and research evidence of intervention effectiveness, yet several participants remain skeptical about these information sources (quote 1). This distrust was interpreted as a barrier to uptake and engagement, with concerns arising about the intervention credibility if the W-MHI was perceived as *‘too cheap’* (quote 2) and not well-known.

Quote 1: *[Regarding W-MHI effectiveness], I think I would take the word of my family and friends, and possibly even GP and therapist, because I’m assuming they have patients that use it as well. But research evidence on the program website itself, […] because they’re picking it, it might not be as reliable.* (Participant 6, female, 21 years of age)

Quote 2: *If I'm doing an online program for 6 weeks, and it costs $20, is this legitimate? Because that seems way too cheap for me. I'm wondering if this is real because that doesn't match up with [typical] therapy prices.* (Participant 17, female, 23 years of age)

Besides, participants lacked trust in W-MHIs because they did not know instructors who worked in the W-MHIs, and that impeded young people’s willingness to engage with online therapist support on the intervention platform. Engagement could be hindered when individuals were hesitant to contact the online support team for assistance (quote 3).

Quote 3: *I trust them [health professionals] that they would use a good framework for you. But if a framework is not working for you, […] if someone is not confident to say anything [to the support team], then I guess it could be difficult, like a big barrier.* (Participant 9, male, 21 years of age)

#### Perceived Need, Motivation, and Commitment to W-MHIs

Nearly one-third of participants became less motivated to complete the entire W-MHI upon noticing an improvement in their mental wellbeing. Individuals with increased self-motivation and self-reliance appeared to have higher engagement with W-MHIs (quote 4). Other facilitators contributing to greater engagement with W-MHIs were young people’s curiosity, willingness to engage, or desire to *‘follow the whole process’* of the intervention.

Quote 4: *I feel that this is something you’ve got to do on your own. You need to really, after a while, got to be independent and start navigating things for yourself and by yourself, and not rely on a therapist.* (Participant 1, female, 18 years of age)

Moreover, perceived need for accessing mental health support and overcoming several barriers associated with in-person therapies (e.g., cost, distance) were noted as facilitators of W-MHI engagement. Yet, a preference to speaking with someone they knew (quote 5) and mental illness symptoms could discourage individuals from engagement (quote 6). Two participants indicated the anticipated consistent commitment from a W-MHI (e.g., described as *‘like factory daily routines’)* as a barrier to their engagement.

Quote 5: *[It wasn’t] meeting my needs […] I wasn’t that satisfied […] Maybe it depends on the situation. […] At some point I stopped [using the program], because I was getting down. I needed to recover faster, and I needed the easiest way to achieve, that was to talk to someone who knows me.* (Participant 7, male, 22 years of age)

Quote 6: *It’s hard to stay committed to the platform. One of the biggest things about depression is a lack of motivation, not really laziness but like sloth behaviour. If you’re having a really bad day, you’re not always going to be motivated to make that effort [to engage with W-MHIs].* (Participant 2, female, 25 years of age)

#### Awareness and Perception of W-MHIs

Individuals mentioned challenges in accessing W-MHIs due to a lack of awareness. Suggestions were made to enhance advertising W-MHIs on online platforms (e.g., YouTube), expanding outreach to young people (quote 7).

Quote 7: *If things were advertised better... I see ads all the time on YouTube for what food you should eat. I rarely see anything for online mental health programs… Unless you’re a student at some institution, I’ve never heard of them outside of it.* (Participant 6, female, 21 years of age)

### Theme 2: Feeling Supported and Willing to Connect

Participants valued the feeling of being supported by others (such as health professionals and other users) and expressed a willingness to connect with them. However, the lack of immediate support from health professionals within W-MHIs could be a barrier.

#### Connecting with Others and Receiving Mental Support

Participants preferred to talk about their mental health problems and get professional advice, thus indicating contact with health professionals as a facilitator of engagement with W-MHIs. Some participants perceived that W-MHIs could be more beneficial with guidance from trained instructors (e.g., health professionals with expertise in this area). However, a negative experience with the service support could be a barrier (quote 8). One participant currently seeing a therapist had expected the lack of health professionals within W-MHIs, and thus their absence was not seen as a barrier.

Quote 8: *If in any way you feel like you were mistreated, you were not given as much attention as you would like […] that could make me stop using the platform all together.* (Participant 14, male, 25 years of age)

In addition, some participants appreciated connecting with other users in W-MHIs, sharing experiences with those having similar challenges and feeling that they were not alone. However, more than half of the participants would not communicate with other users but acknowledged the potential value of this option for other individuals (quote 9).

Quote 9: *It should be fun to meet other people going through the same thing and share ideas and experience. […] I really like it if it can be done that way. [But] it’s not necessarily important.* (Participant 12, male, 22 years of age)

#### Flexible Contact with Health Professionals but Lack of Immediate Responses

In guided W-MHIs with access to health professionals (e.g., therapists), participants appreciated having access to the same therapists and the flexibility to contact therapists (e.g., online). A rigid intervention without the involvement of health professionals could impede participants from expressing their feelings freely, leading to unmet emotional and psychological needs and lower engagement with W-MHIs (quote 10).

Quote 10: *Assessments [within the program] can never be compared [to therapist contact]. They’re never at the same level with a phone call [with therapists] because they give you predetermined possible answers to your question. […] Phone call is actually very important.* (Participant 14, male, 25 years of age)

Young people had varied preferences for contacting therapists. Some liked reaching out to therapists whenever their need arose, while others preferred weekly (some fortnightly) contact for regular progress checking. Most young people favoured more frequent contact, at least twice during the whole intervention. One participant who tended to *‘bottle things up’* preferred monthly contact. Barriers to engagement with online therapist support within W-MHIs included long waiting times and insufficient support during night-time (quote 11).

Quote 11: *At some point it might take time for the therapist to get back to you. That might be the moment I’m willing [to have] someone to talk with. That was one of my barriers.* (Participant 7, male, 22 years of age)

Participants recommended scheduling therapist contact at specific times to mitigate waiting time and increase engagement with W-MHIs. Moreover, participants shared different preferences towards therapist contact approaches (e.g., through an internet chat, audio, or video call), depending on individuals’ comfort level with the respective platforms and their sense of connection with the therapists (quotes 12 and 13).

Quote 12: *Well, I like a video call […] because it allows me to speak freely and express myself. […] I get to see a lot of other things I wouldn’t see [if that’s via chat or phone call], including the body language.* (Participant 13, male, 24 years of age)

Quote 13: *It would be via chat [...] I feel like I can be more open with my emotions and […] I don’t want them to know or see my face in that moment.* (Participant 6, female, 21 years of age)

### Theme 3: Personal Expectation and Experience with W-MHIs

Personal expectation and experience with W-MHIs refers to how well the intervention aligns with users’ needs and expectations. These can include factors related to programme cost, programme features, user interface, and technical aspects of W-MHIs.

#### Programme Cost

Most participants discussed the affordability of W-MHIs, indicating low cost or free access to W-MHIs as a facilitator of engagement. Young people expressed higher willingness to pay if they perceived greater need to use W-MHIs, and found the interventions valuable, especially if therapist contact was included in the interventions (quote 14).

Quote 14: *[Cost] It’s always important. If you’re getting something very valuable, then it’s worth it ... [Something that makes me pay extra], I would say video call [with a therapist] options would be great.* (Participant 14, male, 25 years of age)

Nevertheless, one participant was unwilling to pay for W-MHIs and indicated cost as a barrier (quote 15). Several participants recommended offering upfront payment discounts or instalment plans for paid W-MHIs to mitigate the financial burden associated with these interventions.

Quote 15: *I’m just not going to pay for an online program. It isn’t even face-to-face. It’s an online program. Again, all the factors come in as well, for example, the reputation of the program, how well it is received, how much information it has, and whether it’s really helpful.* (Participant 18, female, 21 years of age)

#### Whether the Programme Meets User Needs

Continued engagement with W-MHIs seemed to depend on the intervention’s ability to meet individuals’ needs and improve their mental wellbeing.

Intervention benefits might be attributed to the relevance and quality of information provided by W-MHIs. The ease of applying knowledge and coping skills to daily lives was reported as a facilitator of engagement with W-MHIs. Nevertheless, heavy content (e.g., involving technical details or requiring effort to understand) was a barrier. Concise, consistent, and relevant content (e.g., tailored to individuals’ specific situations) could facilitate young people’s engagement with W-MHIs. More importantly, dynamic content, preferred by many participants, could actively involve users and encourage return visits if individuals experienced anxiety or depression again. Integration of various components such as brain teasers, meditation, and games was suggested to maintain users’ interest and engagement with W-MHIs (quote 16). Besides, W-MHIs should consider users’ status (new or past) in W-MHIs, allowing past users to re-do lessons or start new ones to avoid potential boredom with previously known content. One participant experiencing both eating disorders and anxiety disengaged from W-MHIs because the intervention was irrelevant to their specific condition and most information was already familiar.

Quote 16: *Another part could be moving away from the angle of therapy as a whole and making it a little bit of activities. Maybe something […] could bring excitement, […], (like) in a child’s case, the volcano game [..]. So, you can come back even when you're not having anxiety. You just want to check it out.* (Participant 14, male, 25 years of age)

Regarding the intervention structure, some participants indicated the fixed lesson order as a barrier to engagement with W-MHIs, while others trusted the design by health professionals. Participants also discussed their preferred number and duration of lessons. Most preferred 6–10 lessons, with each lesson lasting 10–20 min. Two participants were concerned about the potential benefits of W-MHIs with too few lessons (fewer than six), while an excess of lessons (12 or more) and longer lessons (more than 20 min) could make users exhausted, hindering engagement (quote 17). However, some participants preferred spending longer time (from 30 to 45 min, and even more) on each lesson for better absorption of information.

Quote 17: *If you're dealing with sensitive information for a long amount of time, then you could become triggered or sad […]. So, I think around 20 to 30 mins is the maximum, and then you can choose whether you want to spend more time on it … I would want it [the entire program] to be about 6 weeks because I don’t want to commit to something really long term. […] Maybe it’s something that I log on once or twice a week.* (Participant 17, female, 23 years of age)

#### Programme Features

Participants discussed their preferred features in W-MHIs (e.g., initial screening, quizzes, etc.). Many participants found initial screening helpful for determining the intervention’s appropriateness for their current condition. However, a lengthy screening process might discourage users from taking the test and subsequent use of the intervention. Besides, post-lesson quizzes were deemed helpful for validating user’s understanding and fostering a sense of accomplishment, yet a few participants indicated quizzes as a barrier (quote 18). One participant liked the daily user check-in feature, as it made them feel like *‘someone is being cared’*.

Quote 18: *[Quiz] it was helpful. But again, you don't want to do quizzes every single module. You're already doing exams in university, and now you have to do it [the quiz], like an external module.* (Participant 18, female, 21 years of age)

Some participants desired long-term access to the intervention material and suggested a 1-year intervention subscription (quote 19). On the other hand, the majority were uncertain about their future use and would only consider a subscription if it could be shared with their families or friends.

Quote 19: *Yes, to still stay connected with the content in some way, just have it in my memory […] because you tend to forget things. So, you probably need something like to be able to check in, or in some way (to) remain involved.* (Participant 1, female, 18 years of age)

Other facilitators of adopting a W-MHI included intervention name and trial period. A brief trial enabled users to experience the intervention, helping them determine intervention suitability. Participants recommended a concise intervention description (e.g., covering its purposes, funding sources, and testimonials) to provide a broader overview and facilitate uptake of W-MHIs (quote 20).

Quote 20: *A 5-min video is great. It could just talk little bit about people's experiences, and other recovered [from mental illness]. Some people like to see testimonies…. It will help the newcomers make a decision [to use a program].* (Participant 14, male, 25 years of age)

Participants discussed the unpleasant emotions resulting from excessive irrelevant content in W-MHIs (e.g., intrusive advertisements on the screen or *‘bombarded’* via email). Besides, the existence of both free and paid versions of a W-MHI could hinder user engagement due to the concerns about the potential differences in features and content.

#### User Interface

User-friendly interfaces, easy navigation, and attractive layouts were considered as engagement facilitators. Yet, a few participants found the layout *‘not aesthetically pleasing’* and expressed their negative experience with the login page (e.g., it was ‘*reminding of uni work’)*.

It was important to have a variety of tools (e.g., cartoons, videos, and audios) and flexible options to view information (quote 21). All participants appreciated the use of pictures with texts, as *‘a picture [is] worth more than a thousand words’* and could convey information more easily. Videos were largely preferred as they were more engaging and ‘*keep your mind attached’*. However, a few participants expressed privacy concerns, fearing that others *‘can easily tell what I’m doing’* through videos.

Quote 21: *I have a friend who's deaf. She has had so much trouble, especially during the pandemic, in accessing the right technology to help her. Or even if someone was blind, they would need audios. So, I think just having it [texts, pictures, videos, and audios available] as inclusive as possible would be good.* (Participant 17, female, 23 years of age)

#### Technical Aspects

Some participants experienced technical issues, primarily related to failed audio or poor internet connection during web-based sessions. These disruptions were perceived as barriers. Some suggested improving accessibility by allowing users to access the content offline.

### Theme 4: Opportunities and Challenges of Online Platforms

Participants perceived opportunities provided by online platforms, such as accessibility, flexibility, and the ability to remain anonymous, as facilitators of engagement with W-MHIs. However, challenges such as concerns about online security and the inability to replace in-person therapies were regarded as barriers to engagement.

#### Accessibility and Flexibility

Nearly all participants indicated the flexibility and accessibility of W-MHIs as facilitators of engagement. Individuals could conveniently access mental health support at anytime and anywhere, using mobile phones or laptops without travelling to a health professional’s office. Moreover, most participants appreciated the flexibility to pause and resume a specific session at their convenience. Participants had different preferences towards the intervention delivery platform, with some favouring mobile apps for their portability and easy accessibility (e.g., *‘my phone with me 24/7’)*, while others preferred websites for a larger viewing experience and to maintain privacy (e.g., not wanting others to see a mental health app on their phone).

#### Staying Anonymous but Concerns About Online Security

Stigma about mental health was indicated as one of the reasons why participants turned to W-MHIs. More than one-third of participants valued the anonymity for keeping their mental illnesses private due to stigma and they were more willing to seek support by themselves (quote 22). Moreover, stigma appeared to influence how individuals engaged with W-MHIs. For instance, some participants would use *‘private browsing’*, due to fearing that others might know about their use of W-MHIs.

Quote 22: *Most of the times people don’t really talk about things like this [mental health problems]. They actually want to find solutions themselves […] before they actually talk to families and friends, who might give them a recommendation.* (Participant 12, male, 22 years of age)

Many participants were concerned about data privacy associated with online platforms (quote 23). Yet, it was not discussed as a barrier to user engagement, unless W-MHIs required to disclose personal information (e.g., family background, credit card details).

Quote 23: *How my data has been used with (the) third party is also very important […] If the privacy is not secure, then I won’t actually touch that particular program because I’m actually very observant and particular about how much my personal data being handled.* (Participant 12, male, 22 years of age)

#### Cannot Entirely Replace In-Person Therapies

Some limitations of online support were noted as barriers to W-MHIs. One participant feared that chat-based support might not provide the same level of comprehensive care as in-person therapies (quote 24). The absence of human interaction, *‘overlooked body language, facial expressions, and vocal signals’,* was noted as another challenge. Participants recommended incorporating video conferences to overcome this barrier. However, online contact was described as *‘passive interaction’*, especially for those in a severe or crisis condition (quote 25). In addition, online communication sometimes led to miscommunication due to the challenges of fully conveying ideas through texts and pictures.

Quote 24: *It’s only the chat […], it’s just you, sitting in your room, on your phone. When you stop it [the chat], [it] brings up a lot of emotions. […] XX has resources to help you come down healthily, whereas YY, you (kind of) log off, and you don’t have those extra support to come down. That can be very dangerous.* (Participant 2, female, 25 years of age)

Quote 25: *Since the online therapies are distant from clients, it can be very difficult for them to respond quickly and effective. [...] For clients experiencing suicidal thoughts, it can be difficult, even impossible, for the therapist to provide direct assistance. That is quite a challenge.* (Participant 10, male, 23 years of age)

## Discussion

This qualitative study found that barriers and facilitators of user engagement with W-MHIs existed at both individual and intervention levels. At the individual level, greater engagement appeared to be associated with great self-motivation, perceived need for W-MHIs and positive beliefs about W-MHIs. At the intervention level, contact with a health professional within W-MHIs along with rich, relevant, and well-integrated content facilitated engagement, while dissatisfaction with user interface, technical glitches, or excessive advertisements tended to hinder engagement. Failure to meet user needs, particularly among young people with comorbid mental health conditions, was another barrier. All participants valued online platform accessibility and flexibility but were concerned about intervention affordability, privacy, and the absence of face-to-face interaction.

We found that the initial use of W-MHIs was largely influenced by user trust in the intervention potential benefits, which is consistent with the study by O'Connor et al. that identified personal trust as a determinant of engagement with digital health technology [33]. Engagement with W-MHIs could be improved by incorporating research evidence and testimonials from individuals who have benefited from W-MHIs. Our study participants knew about metal health services through campaigns at their universities, or websites, which was consistent with previous research [34]. Several study participants were unaware of alternative W-MHIs, emphasising the need for more effective promotion strategies for W-MHIs, such as launching educational campaigns through various channels (e.g., social media, community events). Targeted marketing on social media can effectively increase individual awareness of mental health and W-MHIs, informing the public about the potential benefits of W-MHIs [35, 36].

Stigma around mental health problems is common among young people and was highlighted in the current study. More than half of young adults believe that mental illness is not accepted by society, contributing to stigma around mental health-related help-seeking [37, 38]. Those experiencing stigma tended to seek help online on their own before sharing their problems with others [16]. We found that the fears of others knowing about their mental illness, as well as treatment access, influenced how young people engaged with W-MHIs. To enhance engagement, future W-MHIs should adapt to multiple platforms (e.g., websites and apps), providing users with flexibility to choose what better suits them. Moreover, it is crucial to reduce stigma and increase awareness of mental health interventions to facilitate help-seeking and use of mental health services in young people [38]. Educational programmes and awareness campaigns (e.g., presentations by those having mental illnesses, advertisements on social media) can be adopted to reduce stigma towards mental illnesses [39, 40]. Notably, mental health-related stigma has been attributed to lower help-seeking behaviour in young males compared with females [41]. Despite various mental health treatment modalities being available, there is an imbalance between the number of males experiencing mental illnesses and those seeking treatment [41]. Despite numerous evidence suggesting that more females experience anxiety and depressive symptoms and females are more likely to seek professional help for mental health concerns compared with males [3], the fact that more males participated in our study may be attributed to Facebook recruitment for mental health research, which has been shown to be an effective strategy for recruiting young males [42]. Moreover, the predominance of male participants in our study may reflect the potential of W-MHIs in overcoming stigma and increasing reach to young people, particularly young males. Further research is needed to explore the impact of W-MHIs on reducing mental health-related stigma among young people.

Positive experience with W-MHIs, largely attributed to the intervention potential benefits, reflects the importance of relevant content. To maintain user engagement, W-MHIs should incorporate short video-based content, feature diverse components (e.g., meditation and game elements), and avoid irrelevant advertisements. Male participants in this study suggested gamification in W-MHIs could foster their engagement, aligning with previous studies demonstrating that gamification could improve motivation and engagement with therapeutic interventions [43]. In addition, we found that contact with a health professional could facilitate engagement with W-MHIs, which is consistent with previous research [44]. Our study participants had different preferences towards contact approaches (e.g., chat, audio call, or video call) and the ability to choose lesson order, depending on the intervention and its framework. While web-based cognitive behavioural therapy interventions (i.e., structured approaches) typically required sequential completion [45], a rigid structure could prevent engagement for some young people. In order to better meet user needs and increase engagement with W-MHIs, future intervention development should offer users some control (e.g., choosing preferred contact method with health professionals or selecting the lesson to start with).

Most study participants expressed a willingness to pay for W-MHIs, particular among those with prior experience with mental health professionals, possibly due to their understanding of the potential benefits of W-MHIs at a relatively low cost. Notably, our study participants were willing to pay more for W-MHIs if they could contact a health professional, which, while enhancing mental health outcomes, requires substantial time and resources [46]. Despite growing research examining the use of chatbots to replace human support in digital interventions [47], these technologies to date lack capacity to simulate empathy relative to human interaction [48]. Future W-MHIs should consider offering health professional contact at an affordable cost to enhance the long-term quality and accessibility of online services.

Despite positive experience with W-MHIs reported by the study participants, it appeared that W-MHIs may not adequately address the needs of young people with comorbidity due to a lack of specialisation in more complex mental health conditions. This limitation could particularly impact individuals with complicated conditions, especially during the COVID-19 pandemic with temporary closures of mental health clinics [49]. While current literature supports the effectiveness of W-MHIs for those with mild anxiety and depression symptoms [50], there is insufficient evidence about W-MHI efficacy in other mental health disorders, such as eating disorders [51]. Providing several barriers to accessing in-person therapies, such as cost and waiting time [16], development of web-based services targeting comorbidity conditions is necessary to expand access and reduce disparities in mental health support.

The strengths of our study include involving end-users of various W-MHIs and exploring factors influencing their engagement (or disengagement) with specific interventions beyond the research context. Our study participants had experience using different types of W-MHIs, including web-based mindfulness interventions, chat support, and structured interventions. Thus, our study can provide a comprehensive picture about barriers/facilitators of W-MHIs. Moreover, participants were recruited online via social media, which has been widely used by young people and described as an effective approach to disseminate mental health-related information to the young population [35, 36]. Furthermore, the qualitative findings were triangulated among the research team to ensure rigour of thematic analysis.

Nevertheless, this study has some limitations. First, some participants used W-MHIs several months before the interview/focus group. It might be challenging to recall the intervention and their experience with it, especially when some had used the intervention more than 6 months ago. Second, the overrepresentation of males in this study may limit the generalisability of the findings to the wider population of young people. Third, participants were self-selected to participate in the interview/focus group and the majority reported positive experience with at least one of the W-MHIs that they have previously used. Individuals with overall negative experience with W-MHIs maybe underpresented in this study, thus it is possible that some barriers were not identified. Lastly, participants were incentivised for their participation, which could potentially introduce bias if participants were motivated primarily by financial reward rather than genuine interest in the study topic. However, our study participants were fully informed about the voluntary nature of their participation. This financial incentive of an AU$20 gift voucher for 1- to 1.5-h interviews/focus groups was deemed appropriate and ethical, as it was below the Australian hourly minimum wage at the time of this study. This incentive aimed to respect participants’ time and effort without coercing their involvement.

## Conclusion

This is the first qualitative study to examine barriers and facilitators of engagement with W-MHIs among young people who are current (or past) intervention users. Barriers at the individual level can be mitigated by adopting mental health promotion strategies and de-stigmatising campaigns to increase the perceived importance of mental health interventions among young people. Health professional contact should be included as part of the W-MHIs. The findings that young people had varied preferences for W-MHIs in terms of programme suitability and usability suggests future W-MHI development should involve young people in the co-design process to enhance user experience and foster their engagement.

## Supplementary Information

Below is the link to the electronic supplementary material.

Supplementary file1 (DOCX 298 KB)

## Declarations

### Data and/or code availability

Supplementary files detail all themes generated with a list of codes. De-identified data may be available upon reasonable request from the corresponding author.
