# Supplementary material for: Young People’s Barriers and Facilitators of Engagement with Web-Based Mental Health Interventions for Anxiety and Depression: A Qualitative Study
Source: Patient. 2024 Jul 13;17(6):697–710. doi: 10.1007/s40271-024-00707-5 (PMC11461805; doi:10.1007/s40271-024-00707-5)
Supplement: Supplementary file 1 — Supplementary file1 (DOCX 298 KB) [file 40271_2024_707_MOESM1_ESM.docx]

# **Supplementary information**

## **Supplementary file 1 – Interview guide**

**Part 1:** Sharing experience of using web-based interventions for anxiety and depression.

1. Describe web-based interventions that you have used to manage your depression/anxiety.
2. Why did you choose (not) to use this intervention?
3. What did you like (or dislike/have concerns) about the intervention? Do you think whether and how these factors influenced your engagement/use of the intervention?
4. If you don’t like [name an intervention aspect], will you come back to use the intervention again?
5. Among those, what is the most important factor that motivated you to use it (or hindered you from using it)?
6. How often did you use the intervention? (prompt: how many times per week)
   - If more than once, what made you come back?
   - Otherwise, why did you stop using it?
7. Have you ever used any other web-based interventions like this? If yes:

- Describe the intervention.
- What did you like and dislike about this intervention?
- Which one did you prefer and why?

1. What additional features would you prefer web-based interventions should have?
2. Would recommend web-based interventions to your friends/peers when they experience stress, depression, or anxiety?
3. Do you have other comments?

**Part 2:** Discuss whether the attributes (and levels) described in Figure S1 below influence your engagement with web-based interventions

**Fig. S1** – A screenshot of all attributes presented to participants during the interview/focus group


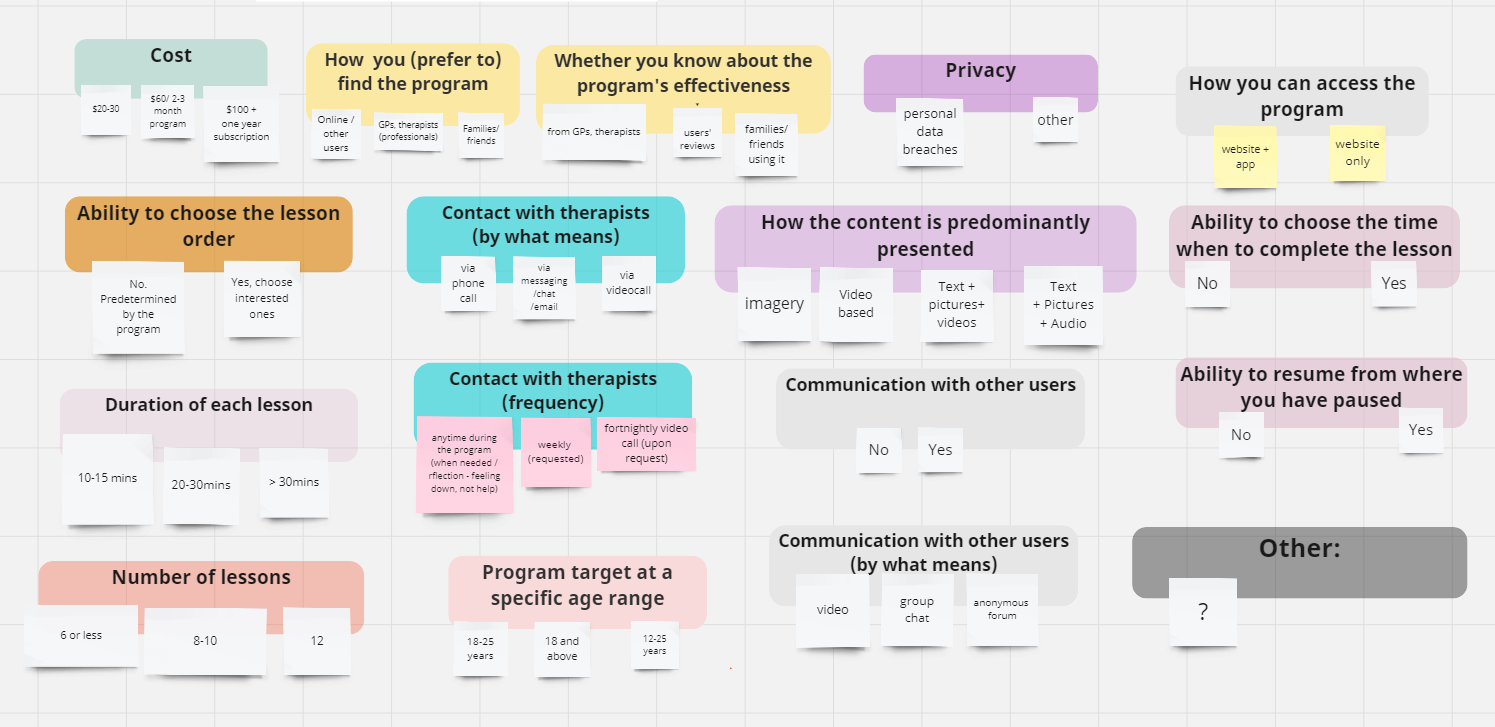


## **Supplementary file 2 – List of codes**

|  | **List of codes** | | | **P1** | **P2** | **P3** | **P4** | **P5** | **P6** | **P7** | **P8** | **P9** | **P10** | **P11** | **P12** | **P13** | **P14** | **P15** | **P16** | **P17** | **P18** | **P19** | **P20** |
| --- | --- | --- | --- | --- | --- | --- | --- | --- | --- | --- | --- | --- | --- | --- | --- | --- | --- | --- | --- | --- | --- | --- | --- |
| 1 | **Theme 1: Intrinsic motivators** | | | | | | | | | | | | | | | | | | | | | | |
| 2 | **1.1.** | **Personal trust in W-MHIs** | | | | | | | | | | | | | | | | | | | | | |
| 3 |  |  | Belief that the program can help me |  |  |  |  |  |  |  |  |  |  |  |  |  | ✓ |  |  |  |  | ✓ |  |
| 4 |  |  | Program is reliable (e.g., government funded) |  | ✓ |  | ✓ |  |  |  |  | ✓ |  |  |  |  |  |  |  | ✓ |  |  | ✓ |
| 5 |  |  | More trust if heard about the program effectiveness |  |  |  |  |  | ✓ |  |  |  |  |  | ✓ | ✓ | ✓ |  |  | ✓ |  |  |  |
| 6 |  |  | *- Not everything works for everyone* | *✓* | *✓* |  |  |  |  |  |  | *✓* |  | *✓* |  |  |  |  |  |  | *✓* |  |  |
| 7 |  |  | More concern (i.e., not using the program) if not referred by anyone |  |  |  |  |  | ✓ |  |  |  |  |  |  |  |  |  |  |  |  |  |  |
| 8 |  |  | More trust if knowing program from trusted ones/previous users; | ✓ |  | ✓ | ✓ | ✓ | ✓ | ✓ | ✓ | ✓ |  | ✓ | ✓ | ✓ | ✓ |  |  |  | ✓ | ✓ |  |
| 9 |  |  | Concerns about reviews/research evidence about W-MHIs | ✓ |  |  |  |  | ✓ |  |  |  |  |  | ✓ |  |  |  |  |  |  |  |  |
| 10 |  |  | Program is popular (e.g., appeared in the top search results) |  |  | ✓ |  |  |  |  |  |  |  |  |  | ✓ |  |  |  |  |  |  |  |
| 11 |  |  | Not knowing who I'm talking to (e.g., they're strangers) |  |  |  |  |  |  | ✓ |  |  |  |  |  |  |  |  |  |  | ✓ |  |  |
| 12 |  |  | Doubts about program if it is free (or too cheap) |  |  |  |  |  |  |  |  | ✓ |  |  |  |  |  |  |  | ✓ |  |  |  |
| 13 | **1.2.** | **Perceived need, motivation, and commitment to W-MHIs** | |  |  |  |  |  |  |  |  |  |  |  |  |  |  |  |  |  |  |  |  |
| 14 |  | **Factors influencing personal growth** | |  |  |  |  |  |  |  |  |  |  |  |  |  |  |  |  |  |  |  |  |
| 15 |  |  | Motivation, dedication, commitment (e.g., you need time to go through it, you need to work on yourself) | ✓ | ✓ | ✓ |  |  |  |  |  |  |  |  |  | ✓ |  | ✓ | ✓ |  | ✓ |  |  |
| 16 |  |  | Barriers if required much commitment or losing motivation due to current condition |  | ✓ |  |  | ✓ |  |  |  |  |  |  |  |  |  |  | ✓ |  | ✓ |  |  |
| 17 |  |  | Curiosity (e.g., want to explore) or instinct/feeling (e.g., want to use) | ✓ |  | ✓ |  |  |  |  |  |  |  | ✓ |  | ✓ | ✓ |  | ✓ |  | ✓ |  | ✓ |
| 18 |  |  | Hesitance (or shyness) to use online support (or talk to therapists online) |  |  |  |  |  |  |  |  | ✓ |  |  |  |  |  |  |  |  |  |  |  |
| 19 |  | **Perceived need for using W-MHIs** | |  |  |  |  |  |  |  |  |  |  |  |  |  |  |  |  |  |  |  |  |
| 20 |  |  | Need arises due to barriers to in-person services (e.g., cost, travel distance, not keeping up appointment) | ✓ | ✓ | ✓ |  |  |  |  |  |  |  |  | ✓ | ✓ | ✓ |  |  | ✓ |  |  |  |
| 21 |  |  | Needs arises due to current condition (e.g., feeling down and need help, stress from work/study) | ✓ |  |  |  |  | ✓ | ✓ |  |  |  | ✓ | ✓ |  |  | ✓ |  |  |  |  | ✓ |
| 22 |  |  | Needs arises due to feeling more comfortable to speak/chat online | ✓ |  |  |  |  |  |  |  |  |  |  |  |  |  |  |  |  |  |  |  |
| 23 |  |  | Not having needs (e.g., not having anxiety at that moment, wanting to talk to someone) |  |  |  |  |  |  | ✓ |  | ✓ |  |  |  |  | ✓ |  |  | ✓ |  |  |  |
| 24 | **1.3.** | **Awareness and perception of mental health and web-based programs** | | | | | | | | | | | | | | | | | | | | | |
| 25 |  |  | Being aware of web-based programs (e.g., seeing ads at uni, searching it online) | ✓ |  |  |  | ✓ | ✓ |  | ✓ | ✓ |  |  | ✓ | ✓ | ✓ |  |  |  |  |  |  |
| 26 |  |  | Lack of awareness about web-based programs (e.g., not knowing about it, having no time to search for other web-based programs) | ✓ |  |  |  |  | ✓ |  |  |  |  |  |  |  |  |  |  |  |  |  |  |
| 27 |  |  | Connotations about online platform (e.g., Zoom is too formal and reminds of Covid-19) |  | ✓ |  |  |  |  |  |  |  |  |  |  |  |  |  |  |  |  |  |  |
| 28 |  |  | Feeling of stigma about my mental health conditions (this influences how I use the program) |  |  |  |  |  | ✓ | ✓ | ✓ | ✓ | ✓ | ✓ |  | ✓ | ✓ | ✓ |  |  |  |  | ✓ |
| 29 |  |  | Not satisfied with previous mental health services |  |  |  |  |  | ✓ |  |  | ✓ |  |  |  |  | ✓ |  |  |  |  |  |  |
| 30 | **Theme 2: Feeling supported and willing to connect** | | | | | | | | | | | | | | | | | | | | | | |
| 31 | **2.1.** | **Connecting with others and receiving mental support** | | | | | | | | | | | | | | | | | | | | | |
| 32 |  |  | Benefits of contacting with health professionals (e.g., getting advice, getting more benefits from experts in the field) | ✓ |  | ✓ | ✓ | ✓ | ✓ | ✓ |  | ✓ |  | ✓ |  | ✓ | ✓ | ✓ | ✓ | ✓ | ✓ | ✓ | ✓ |
| 33 |  |  | Not expecting to have contact with a health professional in a web-based program |  |  |  |  |  |  |  |  |  |  |  |  |  |  |  | ✓ |  |  |  |  |
| 34 |  |  | Communication with other users (e.g., a place to comment, good to have this option but not necessary) |  |  |  |  |  |  | ✓ | ✓ |  |  | ✓ | ✓ | ✓ |  |  | ✓ | ✓ |  | ✓ | ✓ |
| 35 | **2.2.** | **Flexible contact with health professionals but lack of immediate responses** | | | | | | | | | | | | | | | | | | | | | |
| 36 |  |  | Having access to the same therapists |  |  |  | ✓ |  |  |  |  | ✓ |  | ✓ |  | ✓ |  |  |  |  |  |  |  |
| 37 |  |  | Flexibly contacting therapists (i.e., online) |  |  |  |  |  |  |  |  |  |  | ✓ |  |  |  |  |  | ✓ |  | ✓ |  |
| 38 |  |  | *- Preferred frequency of contact* | *✓* | *✓* | *✓* | *✓* | *✓* | *✓* | *✓* | *✓* | *✓* | *✓* | *✓* | *✓* | *✓* | *✓* | *✓* | *✓* | *✓* | *✓* | *✓* | *✓* |
| 39 |  |  | *- Preferred means of contact - depending on my comfort to express feelings on the respective platform* | *✓* | *✓* | *✓* | *✓* | *✓* | *✓* | *✓* | *✓* | *✓* | *✓* | *✓* | *✓* | *✓* | *✓* | *✓* | *✓* | *✓* | *✓* | *✓* | *✓* |
| 40 |  |  | Can tell more and express feelings freely (i.e., not restricted to a rigid program) |  |  |  |  |  |  |  |  | ✓ |  |  |  | ✓ |  |  |  |  |  |  |  |
| 41 |  |  | Limited time to contact therapists is a barrier |  |  |  |  |  |  | ✓ |  |  |  | ✓ |  |  |  |  |  | ✓ |  |  |  |
| 42 |  |  | Waiting time (e.g., therapists are not always available) |  |  |  |  |  | ✓ | ✓ |  | ✓ |  | ✓ |  |  |  |  |  | ✓ |  |  |  |
| 43 | **Theme 3: Personal expectation and experience with web-based programs** | | | | | | | | | | | | | | | | | | | | | | |
| 44 | **3.1.** | **Program cost** | | | | | | | | | | | | | | | | | | | | | |
| 45 |  |  | Free or affordable, worthwhile for what it costs | ✓ |  | ✓ |  |  | ✓ | ✓ | ✓ | ✓ |  | ✓ | ✓ | ✓ | ✓ | ✓ |  | ✓ |  | ✓ | ✓ |
| 46 |  |  | *- Not paying for an online program* |  |  |  |  |  |  |  |  |  |  |  |  |  |  |  |  |  | ✓ |  |  |
| 47 |  |  | Can pay more if there is therapist contact |  |  |  |  |  |  |  |  |  |  |  |  |  |  | ✓ | ✓ |  |  |  |  |
| 48 | **3.2.** | **Whether the program meets users' needs** | | | | | | | | | | | | | | | | | | | | | |
| 49 |  |  | Whether the program is helpful | ✓ | ✓ | ✓ | ✓ |  | ✓ | ✓ | ✓ | ✓ |  | ✓ | ✓ | ✓ | ✓ |  |  | ✓ | ✓ | ✓ | ✓ |
| 50 |  |  | *- Stopping using the program when feeling better* |  |  |  | ✓ | ✓ |  |  |  | ✓ |  |  |  |  | ✓ | ✓ | ✓ |  |  |  |  |
| 51 |  |  | Program content: rich, concise, easy to apply knowledge and coping skills in daily lives, integrating more content/activities, interactive modules |  | ✓ |  |  |  | ✓ |  | ✓ | ✓ |  | ✓ | ✓ | ✓ | ✓ | ✓ | ✓ | ✓ | ✓ |  |  |
| 52 |  |  | Program content: not relevant, not containing many stuffs, mentally heavy, generic, too technical, too many ads/emails, etc.) |  | ✓ |  |  |  | ✓ |  |  | ✓ |  |  | ✓ | ✓ | ✓ |  |  | ✓ | ✓ |  |  |
| 53 |  |  | Suggesting dynamic content |  |  |  |  |  |  |  |  |  |  |  | ✓ | ✓ | ✓ | ✓ | ✓ |  | ✓ |  |  |
| 54 |  |  | Program is relevant and suitable for themselves | ✓ | ✓ | ✓ | ✓ | ✓ | ✓ | ✓ | ✓ | ✓ | ✓ |  | ✓ | ✓ | ✓ | ✓ | ✓ | ✓ | ✓ | ✓ | ✓ |
| 55 |  |  | *- Age-relevance (i.e., program targeting specifically for 18-25yrs, or 18 and above, 13 might be too young)* | ✓ | ✓ |  |  |  |  | ✓ | ✓ |  | ✓ | ✓ | ✓ |  |  |  |  |  |  | ✓ | ✓ |
| 56 |  |  | *- Preferred number of lessons* | ✓ | ✓ | ✓ | ✓ | ✓ | ✓ | ✓ | ✓ | ✓ | ✓ |  | ✓ | ✓ | ✓ | ✓ | ✓ | ✓ | ✓ | ✓ | ✓ |
| 57 |  |  | *- Preferred duration of each lesson (e.g., too long modules will be exhausting)* | ✓ | ✓ | ✓ | ✓ | ✓ | ✓ | ✓ | ✓ | ✓ | ✓ | ✓ | ✓ | ✓ | ✓ | ✓ | ✓ | ✓ | ✓ | ✓ | ✓ |
| 58 |  |  | - *Not the right fit for me (or I'm not eligible for the program) (e.g., due to comorbid eating disorders and anxiety/depression)* |  |  |  |  |  |  |  |  |  |  |  |  |  |  |  |  | ✓ |  |  |  |
| 59 |  |  | Ability to choose the lesson order |  |  |  |  |  | ✓ | ✓ | ✓ | ✓ |  | ✓ |  |  |  |  | ✓ | ✓ |  | ✓ |  |
| 60 | **3.3.** | **Program features** | | | | | | | | | | | | | | | | | | | | | |
| 61 |  |  | Access in the long term, on the regular basis / Might not use again in the long term | ✓ |  |  |  |  | ✓ | ✓ |  |  |  | ✓ | ✓ | ✓ | ✓ |  | ✓ | ✓ |  |  |  |
| 62 |  |  | Quizzes - helpful, but some dislike it | ✓ |  |  | ✓ |  |  |  |  | ✓ |  |  |  | ✓ | ✓ | ✓ | ✓ |  | ✓ |  |  |
| 63 |  |  | Short program trial (as it can build trust in the program) |  |  |  |  |  |  |  |  |  |  |  | ✓ |  |  |  |  |  |  |  |  |
| 64 |  |  | Dissatisfied with the presence of two program options (free/paid) |  |  |  |  |  |  |  |  |  |  |  |  |  | ✓ |  |  |  |  |  |  |
| 65 |  |  | Initial assessment - helpful but can be too long |  |  |  | ✓ |  |  |  |  |  |  |  |  |  | ✓ |  | ✓ |  |  |  |  |
| 66 |  |  | Abstractive description on the first page |  |  |  |  |  |  |  | ✓ |  |  |  |  |  |  | ✓ | ✓ |  | ✓ |  |  |
| 67 |  |  | Daily check-ins |  |  |  | ✓ |  |  |  |  |  |  |  |  |  |  |  |  |  |  |  | ✓ |
| 68 |  |  | Videos showing testimonies |  |  |  |  | ✓ |  |  |  |  |  |  |  |  | ✓ |  |  |  |  |  |  |
| 69 |  |  | Progress tracking |  |  |  |  | ✓ |  |  |  |  |  |  |  |  |  |  |  |  |  |  |  |
| 70 |  |  | Program name sounds cool |  |  |  |  |  | ✓ |  | ✓ |  |  |  |  |  |  |  |  |  |  |  |  |
| 71 | **3.4.** | **User interface** | | | | | | | | | | | | | | | | | | | | | |
| 72 |  |  | User friendly, easy navigation | ✓ | ✓ | ✓ |  | ✓ |  | ✓ | ✓ | ✓ |  |  | ✓ | ✓ | ✓ |  | ✓ | ✓ | ✓ |  | ✓ |
| 73 |  |  | Nice, attractive layout |  |  |  |  |  |  | ✓ | ✓ | ✓ |  | ✓ | ✓ | ✓ | ✓ |  |  |  |  |  | ✓ |
| 74 |  |  | Not very easy to use (e.g., you have to find around a couple of things) |  |  |  |  |  | ✓ | ✓ |  |  |  |  |  |  | ✓ |  |  |  |  | ✓ |  |
| 75 |  |  | Not engaging |  | ✓ |  |  |  | ✓ |  |  |  |  |  |  |  |  |  |  |  |  | ✓ |  |
| 76 |  |  | Content presentation (e.g., text, audio, video, emoji) | ✓ | ✓ | ✓ |  |  | ✓ |  | ✓ | ✓ | ✓ | ✓ | ✓ | ✓ | ✓ | ✓ | ✓ | ✓ | ✓ | ✓ |  |
| 77 |  |  | *- Just text* |  |  |  |  |  | *✓* |  |  |  |  |  |  |  |  |  |  |  |  |  |  |
| 78 |  |  | *- Like having audio-based content* | *✓* | *✓* |  |  |  |  |  | *✓* |  |  | *✓* | *✓* |  |  | *✓* | *✓* | *✓* |  |  |  |
| 79 |  |  | *- Like having have video-based content* |  | *✓* | *✓* |  |  |  |  |  | *✓* | *✓* |  | *✓* | *✓* | *✓* | *✓* | *✓* |  | *✓* | *✓* |  |
| 80 |  |  | *- Emoji / Cartoon / Pictures* | *✓* | *✓* |  |  |  | *✓* |  | *✓* |  |  |  | *✓* | *✓* |  |  |  |  |  |  |  |
| 81 |  |  | *- Versatility (e.g., I can choose either to read or listen)* |  |  |  |  |  |  |  |  |  |  |  |  |  |  | ✓ |  |  |  |  |  |
| 82 | **3.5.** |  | **Technical aspects** (e.g., Internet connection, program not loading, audio not working) |  |  |  | ✓ |  |  |  | ✓ | ✓ | ✓ | ✓ | ✓ |  |  |  |  |  |  | ✓ | ✓ |
| 83 | **Theme 4: Opportunities and challenges of online platforms** | | | | | | | | | | | | | | | | | | | | | | |
| 84 | **4.1.** | **Accessibility and flexibility** | | | | | | | | | | | | | | | | | | | | | |
| 85 |  |  | Can access via laptop/mobile phone (mixed preferences regarding the use of apps/website) |  |  |  |  |  | ✓ | ✓ | ✓ | ✓ | ✓ | ✓ | ✓ | ✓ | ✓ | ✓ | ✓ | ✓ | ✓ | ✓ | ✓ |
| 86 |  |  | *- Versatility - Consider as barrier if the program restricts the access from one platform (i.e., either mobile phone, or laptop)* |  |  |  |  |  |  | *✓* | *✓* |  |  |  | *✓* |  | *✓* | *✓* |  |  |  |  | *✓* |
| 87 |  |  | *- Use a website/app due to privacy* |  |  |  |  |  |  | *✓* |  |  |  | *✓* |  | *✓* |  |  |  |  |  |  |  |
| 88 |  |  | *- Use a website/app due to convenience/portability* |  |  |  |  |  | *✓* | *✓* | *✓* | *✓* |  |  | *✓* |  |  |  |  |  |  |  |  |
| 89 |  |  | Can access at any time at my own comfort | ✓ | ✓ | ✓ |  | ✓ | ✓ |  |  | ✓ |  | ✓ | ✓ | ✓ | ✓ | ✓ |  | ✓ |  | ✓ | ✓ |
| 90 |  |  | Flexible to pause or resume the sessions |  |  | ✓ |  |  | ✓ | ✓ | ✓ | ✓ | ✓ | ✓ | ✓ | ✓ | ✓ | ✓ | ✓ | ✓ | ✓ | ✓ | ✓ |
| 91 | **4.2.** | **Staying anonymous but concerns about online security** | | | | | | | | | | | | | | | | | | | | | |
| 92 |  |  | Want to keep things private / Maintain privacy |  | ✓ |  |  |  | ✓ | ✓ | ✓ | ✓ | ✓ | ✓ | ✓ |  |  |  |  | ✓ |  | ✓ | ✓ |
| 93 |  |  | *- Concern that others know I'm using these programs* |  |  |  |  |  | ✓ |  |  |  | ✓ |  |  | ✓ | ✓ | ✓ |  |  |  |  |  |
| 94 |  |  | No concern about data breach, yet worry if being asked about personal details |  |  | ✓ | ✓ | ✓ |  |  |  |  | ✓ |  |  |  | ✓ | ✓ | ✓ |  | ✓ | ✓ | ✓ |
| 95 |  |  | Concern about data breach |  | ✓ |  |  |  |  |  |  |  | ✓ |  | ✓ | ✓ |  |  |  |  |  |  |  |
| 96 | **4.3.** | **Cannot entirely replace in-person therapies** | | | | | | | | | | | | | | | | | | | | | |
| 97 |  |  | Challenges of no human interactions (e.g., hard to explain things online, lack of body language) |  |  |  | ✓ |  | ✓ | ✓ |  | ✓ |  |  |  |  |  |  |  | ✓ | ✓ |  | ✓ |
| 98 |  |  | Online interaction is passive (i.e., cannot actively help in the critical situation) |  |  | ✓ |  |  |  |  |  |  | ✓ |  |  |  |  |  |  |  |  |  |  |
| 99 |  |  | Face-to-face support is important (e.g., chat-based can't be the same as in-person therapies) |  | ✓ |  | ✓ |  |  | ✓ |  |  |  |  |  |  |  |  |  | ✓ |  |  |  |

| **Data saturation checking:** | **N** | **%** |  |  |  |  |  |  | New code emerged after 8 interviews |
| --- | --- | --- | --- | --- | --- | --- | --- | --- | --- |
| Total number of codes/sub-codes identified after 8 interviews | 69 | 85.2% |  |  |  |  |  |  | New subcode emerged after 8 interviews |
| Total number of codes/sub-codes identified after 13 interviews | 75 | 92.6% |  |  |  |  |  |  |  |
| Total number of codes/sub-codes identified after 17 interviews | 80 | 98.8% |  |  |  |  |  |  |  |
| Total number of codes/sub-codes identified after 18 interviews | 81 | 100.0% |  |  |  |  |  |  |  |
| **Total number of codes/sub-codes identified after 20 interviews** | **81** | **100.0%** |  |  |  |  |  |  |  |
